# Accuracy of clinicians’ ability to predict the need for renal replacement therapy: a prospective multicenter study

**DOI:** 10.1186/s13613-022-01066-w

**Published:** 2022-10-15

**Authors:** Alexandre Sitbon, Michael Darmon, Guillaume Geri, Paul Jaubert, Pauline Lamouche-Wilquin, Clément Monet, Lucie Le Fèvre, Marie Baron, Marie-Line Harlay, Côme Bureau, Olivier Joannes-Boyau, Claire Dupuis, Damien Contou, Virginie Lemiale, Marie Simon, Christophe Vinsonneau, Clarisse Blayau, Frederic Jacobs, Lara Zafrani

**Affiliations:** 1 Médecine Intensive et Réanimation, Hôpital Saint-Louis, Assistance Publique-Hôpitaux de Paris (AP-HP) Nord, 1 Avenue Claude Vellefaux, 75010 Paris, France; 2 Sorbonne Université, Paris, France; 3 Médecine Intensive et Réanimation, Hôpital Ambroise Paré, Assistance Publique-Hôpitaux de Paris (AP-HP) Sud, Boulogne Billancourt, France; 4 Médecine Intensive et Réanimation, Hôpital Cochin, Assistance Publique-Hôpitaux de Paris (AP-HP) Sud, Paris, France; 5 Médecine Intensive et Réanimation, CHU de Nantes, Nantes, France; 6 Département d’Anesthésie-Réanimation, Hôpital St-Eloi, CHRU, Montpellier, France; 7 Médecine Intensive et Réanimation, Hôpital Bichat, Assistance Publique-Hôpitaux de Paris (AP-HP) Nord, Paris, France; 8 Réanimation Polyvalente, Centre Hospitalier du Sud-Francilien, Corbeil-Essonnes, France; 9 Médecine Intensive et Réanimation, CHU Hautepierre, Strasbourg, France; 10 Médecine Intensive et Réanimation, Hôpital de La Pitié-Salpêtrière, Assistance Publique-Hôpitaux de Paris (AP-HP), Sorbonne Université, Paris, France; 11 Département d’Anesthésie-Réanimation Sud, Centre Médico-Chirurgical Magellan, Bordeaux, France; 12 Médecine Intensive et Réanimation, CHU Gabriel Montpied, Clermont-Ferrand, France; 13 Réanimation Polyvalente, CH Victor Dupouy, Argenteuil, France; 14 Médecine Intensive et Réanimation, CHU Edouard Herriot, Lyon, France; 15 Réanimation Polyvalente, Hôpital de Béthune Beuvry, Béthune, France; 16 Médecine Intensive et Réanimation, Hôpital Tenon, Assistance Publique-Hôpitaux de Paris (AP-HP), Sorbonne Université, Paris, France; 17 Médecine Intensive et Réanimation, Hôpital Antoine Béclère, Assistance Publique-Hôpitaux de Paris (AP-HP), Clamart, France; 18 Université Paris Cité, Paris, France

**Keywords:** Renal replacement therapy, Physician prediction, Acute kidney injury, Intensive care unit

## Abstract

**Purpose:**

Identifying patients who will receive renal replacement therapy (RRT) during intensive care unit (ICU) stay is a major challenge for intensivists. The objective of this study was to evaluate the performance of physicians in predicting the need for RRT at ICU admission and at acute kidney injury (AKI) diagnosis.

**Methods:**

Prospective, multicenter study including all adult patients hospitalized in 16 ICUs in October 2020. Physician prediction was estimated at ICU admission and at AKI diagnosis, according to a visual Likert scale. Discrimination, risk stratification and benefit of physician estimation were assessed. Mixed logistic regression models of variables associated with risk of receiving RRT, with and without physician estimation, were compared.

**Results:**

Six hundred and forty-nine patients were included, 270 (41.6%) developed AKI and 77 (11.8%) received RRT. At ICU admission and at AKI diagnosis, a model including physician prediction, the experience of the physician, SOFA score, serum creatinine and diuresis to determine need for RRT performed better than a model without physician estimation with an area under the ROC curve of 0.90 [95% CI 0.86–0.94, *p* < 0.008 (at ICU admission)] and 0.89 [95% CI 0.83–0.93, *p* = 0.0014 (at AKI diagnosis)]. In multivariate analysis, physician prediction was strongly associated with the need for RRT, independently of creatinine levels, diuresis, SOFA score and the experience of the doctor who made the prediction.

**Conclusion:**

As physicians are able to stratify patients at high risk of RRT, physician judgement should be taken into account when designing new randomized studies focusing on RRT initiation during AKI.

**Supplementary Information:**

The online version contains supplementary material available at 10.1186/s13613-022-01066-w.

## Introduction

Acute kidney injury (AKI) occurs in up to 50% of critically ill patients and is associated with increased mortality and morbidity [1]. Among them, 5% will receive renal replacement therapy (RRT) [2–4]. The optimal timing to initiate RRT has led to multiple randomized trials [5, 6]. According to these trials, except in patients with life-threatening complications of uremia (e.g., severe acidosis, hyperkalemia, severe intoxication, pulmonary edema due to fluid overload), there is no benefit of early RRT initiation in patients with intermediate risk of receiving RRT [7].

The diagnosis of AKI currently relies on serum creatinine elevation or oliguria [8]. However, oliguria is a non-specific marker and serum creatinine elevation is often delayed even when renal damages are already installed [9–11]. Early detection of kidney injury is therefore crucial, in order to diagnose AKI and identify patients who will receive RRT.

Numerous urine and plasma biomarkers have been proposed to predict short and long-term prognosis of AKI among which interleukin-18 (IL-18), cystatine C, neutrophil gelatinase-associated lipocalin (NGAL), kidney injury molecule-1 (KIM-1) or Nephrocheck^™^, which is the product of tissue inhibitor of metalloproteinase-2 (TIMP-2) and insulin-like growth factor-binding protein-7 (IGFBP7). However, none of them have shown sufficient accuracy to predict the need for RRT at bedside [12, 13]. Similarly, Doppler-based resistive index have failed to distinguish patients with transient AKI from those with persistent AKI [14–16].

The furosemide stress tests has also been proposed to predict the progression of AKI but requires the use of a therapy with potential side effects, especially in critically ill patients with hemodynamic instability [17, 18].

Some authors have shown that the combination of clinical models with various biomarkers provides a better ability to predict outcomes [19]. However, in most of these studies, the accuracy of clinician’s ability to predict the need for RRT was not taken into account.

Interestingly, Darmon et al. reported in a multicenter study focusing on the performance of Doppler-based resistive index and semi-quantitative renal perfusion in predicting persistent AKI that clinician’s prediction of probability for short-term renal recovery at study inclusion had moderate-to-good performance in predicting persistent AKI or need for RRT [14]. Nevertheless, data focusing on accuracy of clinician’s estimations are lacking. Clinicians’ abilities to discriminate between patients who will or will not receive RRT are important for several reasons. First, knowledge of future renal function may be very important to ICU patients, their families and physicians. This is particularly true as increasing numbers of AKI patients survive the ICU but experience long-term renal sequelae. Second, predictions of future kidney function may influence clinician behavior, as physicians are more likely to offer the withdrawal of life support when they believe the patient will experience multiple organ dysfunctions.

The objective of this study is to evaluate the performance of physicians in predicting the need of RRT at intensive care unit (ICU) admission and at AKI diagnosis in critically ill patients.

## Material and methods

“PresagEER” study was a prospective, observational, French, multicenter study.

This study was conducted during 3 weeks from October 5, 2020 to October 26, 2020 in 16 ICUs in France. This study was approved by the “Société de reanimation de langue française” (SRLF) ethics committee (CE SRLF 19–30). The study is registered in the INDS study directory under the MR-004 format (n° MR3818070920). According to the French regulation, the need for informed consent was waived. Patients were informed that their data may be used for research purposes and none refused. The study was conducted in accordance with the Declaration of Helsinki principles.

## Clinician study population

The intensivist’ opinion on the likelihood of using RRT was sought at ICU admission and at AKI diagnosis using a visual Likert scale ranging from 0 (“the patient will not require RRT during ICU stay”) to 10 (certainty of the clinician that the patient will require RRT) (Additional file 1: Fig. S1). Surveys were distributed to each investigator of the participating ICUs. We recorded clinician's experience in the ICU (< 2 years, 2–5 years, 5–10 years or > 10 years). In total, 49 (48%) attendings and 54 (52%) fellows completed the surveys. The median number of predictions per physician at admission was 3 [1–7] patients. The physicians who completed the surveys had access to clinical and biological data of the patients, but were different from those who cared for patients and decided for RRT initiation at any time during the study. The survey was completed at three different time points (upon admission (time 1), at AKI diagnosis (the day of AKI diagnosis) in case of AKI occurrence (time 2) and at ICU discharge (time 3) (Additional file 1: Fig. S2). The decision of RRT initiation was left to the discretion of the physician in charge of the patient.

## Patient cohort and data collection

All consecutive patients aged ≥ 18 years admitted to ICU were included. Patients who were under the age of 18 years, had end-stage chronic kidney disease with dependency on RRT, or were pregnant were excluded. Sequential Organ Failure Assessment (SOFA) score was recorded at ICU admission, at AKI diagnosis and at ICU discharge, as previously described [20].

Patient’s medical history was recorded including chronic renal failure, baseline serum creatinine, baseline glomerular filtration rate (GFR) (ml/min) according to the Chronic Kidney Disease-Epidemiology collaboration (CKD-EPI) formula, chronic heart failure, hypertension, chronic respiratory failure, diabetes mellitus, chronic liver failure, immunosuppressive disorders, active smoking, chronic exogenous disease. Serum creatinine level, uremia, urinary output of the last 24 h and fluid balance of the last 24 h were collected at each time point.

Causes of AKI were classified as pre-renal, intra-renal and post-renal (or obstructive) causes [21]. The use of nephrotoxic drugs before the occurrence of AKI was recorded (including aminoglycosides, vancomycin, nephrotoxic chemotherapy, calcineurin inhibitors, angiotensin-converting enzyme inhibitors (ACEIs) or angiotensin receptor blocker (ARB) therapy, non-steroidal anti-inflammatory drugs (NSAIDs), intravenous iodinated contrast media). Mechanical ventilation, extracorporeal membrane oxygenation (ECMO), use of vasopressors, sodium bicarbonates or diuretics were also recorded.

At ICU discharge, need for RRT and modalities of RRT, including date of initiation, dependence on RRT at discharge, date of last RRT session, date of diuresis recovery (> 0.5 ml/kg/h), use of continuous veno-venous hemofiltration (CVVHF) or intermittent hemodialysis (IHD) or sustained low-efficiency dialysis (SLED) were recorded.

Reasons for need of RRT were collected including hyperkalemia, metabolic acidosis, fluid overload, tumor lysis syndrome, oligo-azotemia and/or oliguria.

This is an observational study, so there were no recommendations given to the physicians on when and why they should start RRT. However, all patients who fulfilled the AKIKI “late criteria” received dialysis the same day (including blood urea nitrogen level higher than 40 mmol/l, a serum potassium concentration greater than 6 mmol/l, a pH below 7.15, and acute pulmonary edema due to fluid overload responsible for severe hypoxemia despite diuretic therapy) [22].

Finally, ICU mortality and decisions to withdraw life-sustaining therapies were recorded.

## Definitions

AKI was defined according to Kidney Disease: Improving Global Outcomes (KDIGO) criteria [8]. AKI stage 1 is characterized by an increase in serum creatinine of ≥ 0.3 mg/dl or 1.5 to 1.9 times baseline or urine output of < 0.5 ml/kg/h for 6 to 12 h. AKI stage 2 by increase in serum creatinine to 2.0 to 2.9 times baseline or urine output of < 0.5 ml/kg/h for 12 to 24 h. AKI stage 3 is defined by increase in serum creatinine to ≥ 3.0 times baseline or increase in serum creatinine of ≥ 0.3 mg/dl to ≥ 4.0 mg/dl or urine output of < 0.3 ml/kg/h for ≥ 24 h or anuria for ≥ 12 h or initiation of renal replacement therapy. Basal serum creatinine was defined as the serum creatinine measured in the 3 months preceding the hospitalization in the ICU or, in case of missing data, we used the back-calculation serum creatinine according to the Modification of Diet in Renal Disease (MDRD) formula, assuming a normal GFR of 75 ml/min, as previously described [23].

## Endpoints

The primary endpoint of this study was to evaluate physicians’ performance in predicting the need for RRT at ICU admission (time 1).

Secondary endpoints were to assess physicians’ performance in predicting the use of RRT at AKI diagnosis (time 2), to assess factors associated with clinician judgement and to develop a model able to stratify the risk of RRT in ICU patients.

## Statistical analysis

Data were described as median and interquartile range (IQR) or number and percentage. Categorical variables were compared using Fisher's exact test and continuous variables using the nonparametric Wilcoxon test, Mann–Whitney test, or Kruskal–Wallis test.

It was pre-planned to assess diagnostic performance of physician perception as continuous variables.

To assess discrimination, physician perception of RRT risk were plotted against subsequent need for RRT as the receiver-operating characteristic (ROC) curves of the proportion of true positives against the proportion of false positive to classify patients. Confidence interval of AUC was calculated and AUROC curves compared according to the DeLong method [24]. Sensitivity and specificity confidence intervals were approximated using bootstrapping methods [25, 26]. Optimal cut-point, corresponding to the cut-off on the visual Likert scale with the best sensitivity and specificity, was defined according to optimal Youden’s J statistic [27]. For better readability, the optimal cut-point has been expressed in percentage in the manuscript.

AUC of ROC curves were compared using DeLong methods [24]. AUC of ROC curves were performed first without clinician assessment and then with clinician assessment.

To assess risk stratification of physician perception, we first developed mixed logistic regression model of variables associated with risk of receiving RRT. We used conditional stepwise regression with 0.2 as the critical *P*-value for entry into the model, and 0.1 as the *P*-value for removal. To account for clustering by attending intensivist, intensivist making prediction in the study was included in the model as random effect against the intercept. The variable of interest was need for RRT. First, a model of variables associated without physician perception was built. Then physician perception at admission and AKI onset were forced one by one. Interactions and correlations between the explanatory variables were carefully checked. Continuous variables for which log-linearity was not confirmed were transformed into categorical variables according to median or IQR. The final models were assessed by calibration, discrimination and relevancy. Residuals were plotted, and the distributions inspected. Discrimination of models were plotted and compared.

All tests were two-sided, and *P*-values less than 0.05 were considered statistically significant. Analyses were done using R software version 3.4.4 (https://www.r-project.org), including ‘pROC’, ‘lme4’ and ‘lmerTest’ packages.

## Results

### Patients’ characteristics and outcomes

Six hundred forty-nine patients were included in the study during the inclusion period.

The clinical and biological characteristics of the patients are presented in Table 1. Among the 649 patients included, 70% were men with a median age of 64 [53–73] years. Five hundred and ninety-eight patients (92%) were hospitalized for medical reasons.

**Table 1:** Characteristics of patients, physician prediction and outcomes

Characteristics	No RRT (*n* = 572)	RRT (*n* = 77)	Overall (*n* = 649)	*P* value
Age—years (median [IQR])	64 [52, 73]	64 [54, 72]	64 [53, 73]	0.83
Female gender (%)	177 (31)	20 (26)	198 (30.4)	0.443
BMI (median [IQR])	26.50 [23, 31]	27 [24, 32]	27 [23, 31]	0.063
CCI (median [IQR])	3 [2, 5]	4 [3, 6]	4 [2, 6]	0.001
Charlson score without age (median [IQR])	1 [0, 3]	2 [1, 4]	1 [0, 3]	< 0.001
SOFA score-ICU admission (median [IQR])	4 [2, 7]	7 [5, 12]	4 [3, 8]	< 0.001
Comorbidity (%)				
CKD	56 (9.8)	18 (23.4)	74 (11.4)	0.001
Congestive heart failure	84 (14.7)	15 (19.7)	99 (15.3)	0.33
Myocardial infarction	68 (11.9)	9 (11.7)	77 (11.9)	1
Diabetes mellitus	137 (24)	27 (35.1)	164 (25.3)	0.049
Peripheral vascular disease	59 (11.2)	13 (17.6)	72 (12)	0.163
Chronic pulmonary disease	102 (17.9)	10 (13)	112 (17.3)	0.367
Connective tissue disease	15 (2.8)	3 (4.1)	18 (3.0)	0.836
Liver disease	58 (10.1)	16 (20.8)	74 (11.4)	0.01
Hematological disease	31 (5.4)	8 (10.4)	39 (6.0)	0.142
Metastatic solid tumor	23 (4.4)	3 (4.1)	26 (4.3)	1
AIDS	14 (2.7)	3 (4.1)	17 (2.8)	0.761
ICU: reasons for admission (%)				0.115
Medical causes	527 (92.3)	71 (92.2)	598 (92.3)	
Elective surgery	27 (4.7)	1 (1.3)	28 (4.3)	
Emergency surgery	17 (3)	5 (6.5)	22 (3.4)	
COVID-19 patients (%)	212 (37.2)	30 (39)	242 (37.4)	0.861
Data’s at ICU admission (median [IQR])				
Serum creatinine—µmol/l	79 [61, 116]	166 [85, 250]	82 [63, 134]	< 0.001
Urinary output—ml/kg/h	0.8 [0.4, 1.3]	0.5 [0.3, 1.1]	0.7 [0.4, 1.3]	0.002
Fluid balance—ml/h	0.0 [−34, 58]	44 [0.0, 125]	0.0 [−298, 66]	< 0.001
Fluid balance—ml/kg/h	0.1 [−0.6, 1.0]	0.8 [0.3, 1.8]	0.2 [−0.5, 1.1]	< 0.001
Physician’s ICU experience				0.018
< 2 years	116 (20.3)	26 (33.8)	142 (21.9)	
[2–5] years	148 (25.9)	13 (16.9)	161 (24.8)	
[5–10] years	168 (29.4)	16 (20.8)	184 (28.4)	
> 10 years	140 (24.5)	22 (28.6)	162 (25)	
Physician’s ICU seniority				0.535
Fellows	264 (46.2)	39 (50.6)	303 (46.7)	
Attendings	308 (53.8)	38 (49.4)	346 (53.3)	
Prediction to need of RRT (median [IQR])	1 [0, 3]	7 [4, 10]	2 [0, 4]	< 0.001
Outcomes at discharge				
Death during ICU stay (%)	85 (14.9)	48 (62.3)	133 (20.5)	< 0.001
Time from admission to death—days (median [IQR])	6 [2, 17.5]	13 [5, 22]	9 [3, 20]	0.024
ICU duration—days (median [IQR])	4 [2, 9]	13 [5, 21]	5 [2, 10]	< 0.001
Serum creatinine at ICU discharge—µmol/l (median [IQR])	68 [52, 101]	186 [107, 295]	72 [53, 115]	< 0.001
Urinary output at ICU discharge—ml (median [IQR])	1600 [1080, 2278]	138 [0, 925]	1457 [800, 2200]	< 0.001

*BMI* body mass index, *CCI* Charlson comorbidity index, *SOFA* Sequential Organ Failure Assessment, *CKD* chronic kidney disease, *AIDS* acquired immuno-deficiency syndrome, *ICU* intensive care units, *RRT* renal replacement therapy

Two hundred and forty-two patients (37%) were tested positive for SARS-COV2 during the inclusion period. The median SOFA score at admission was 4 [3–8].

Two hundred and seventy (42%) patients developed AKI. According to the KDIGO score, 114 patients (42%) had AKI stage I, 75 (28%) AKI stage II and 81 (30%) AKI stage III. Etiologies of AKI as perceived by physician were obstructive in 7 (3%) patients, pre-renal in 216 (80%) patients, and intra-renal in 92 (34%) patients. Fifty-eight (21%) patients had mixed causes of AKI. Seventy-seven patients (29% of AKI patients) received RRT during ICU stay. One hundred and forty-six patients (54.1% of patients who developed AKI) had AKI at admission. This represents 22.5% of the entire cohort. The median delay to develop AKI from ICU admission was 0 [0–2] days (Additional file 1: Table S1).

Among the 77 patients who received RRT, 49 (64%) were still dependent on RRT at ICU discharge. At RRT initiation, 13(16.8%) had hyperkalemia > 6 mmol/l, 7 patients (9.1%) had hyperphosphatemia > 3 mmol/l, 14 (18.1%) patients had a pH below 7.15, and 30 (39.5%) patients had fluid overload responsible for severe hypoxemia. Eighteen (23.4%) patients had at least two of these conditions. Characteristics of RRT are shown in Additional file 1: Table S2.

One hundred and thirty-three patients (20.5%) died in ICU and the median length of stay in ICU was 5 [2–10] days.

The patients who received RRT had significantly higher ICU mortality rates than those who did not received RRT (*p* < 0.001) and had significantly higher median length of stay in ICU than those who did not received RRT (*p* < 0.001). Characteristics of patients at AKI diagnosis are shown in Additional file 1: Table S3.

### Discriminative accuracy of physician prediction in predicting RRT requirement

At ICU admission, physicians estimated risk of receiving RRT at 7 [4–10] for patients who ultimately received RRT vs 1 [0–3] in those who did not ultimately receive RRT (*p* < 0.001). At AKI diagnosis, the prediction score was 9 [6–10] for patients who received finally RRT and 3 [1–6] for those did not ultimately received during ICU (*p* < 0.001).

**Fig. 1:**
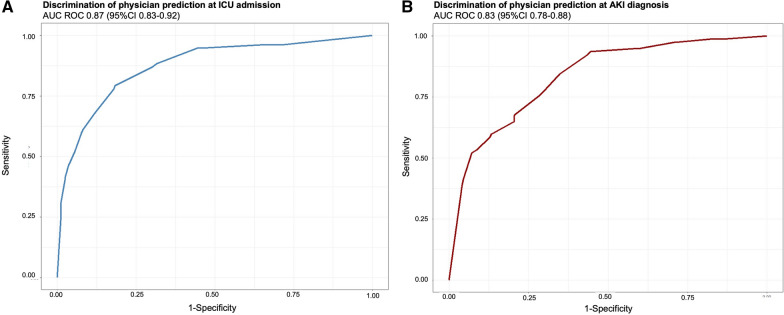
Discrimination of physician prediction at ICU admission (**A**) and AKI diagnosis (**B**). *ICU*   intensive care unit, *AKI * acute kidney injury

Figure 1A and B describes discrimination of physician prediction in predicting need for RRT at ICU admission (Fig. 1A) and at AKI diagnosis (Fig. 1B). Performance of physician were good with area under the ROC curve (AUC) at 0.87 (95% CI 0.83–0.92) at ICU admission and an AUC at 0.83 (95% CI 0.78–0.88) at AKI diagnosis.

For physician perception at ICU admission, the optimal cut-off was 32.5%, with a sensitivity and a specificity of, respectively, 79.2% (95% CI 70.1%-88.3%) and 81.6% (95% CI 78.5–84.8%).

For physician perception at AKI onset, the cut-off was of 40%, with a sensitivity and specificity of, respectively, 84.4% (95% CI 76.6%-92.2%) and 65.1% (95% CI 58.4–71.8%).

### Risk stratification of physician perception at ICU admission

**Fig. 2:**
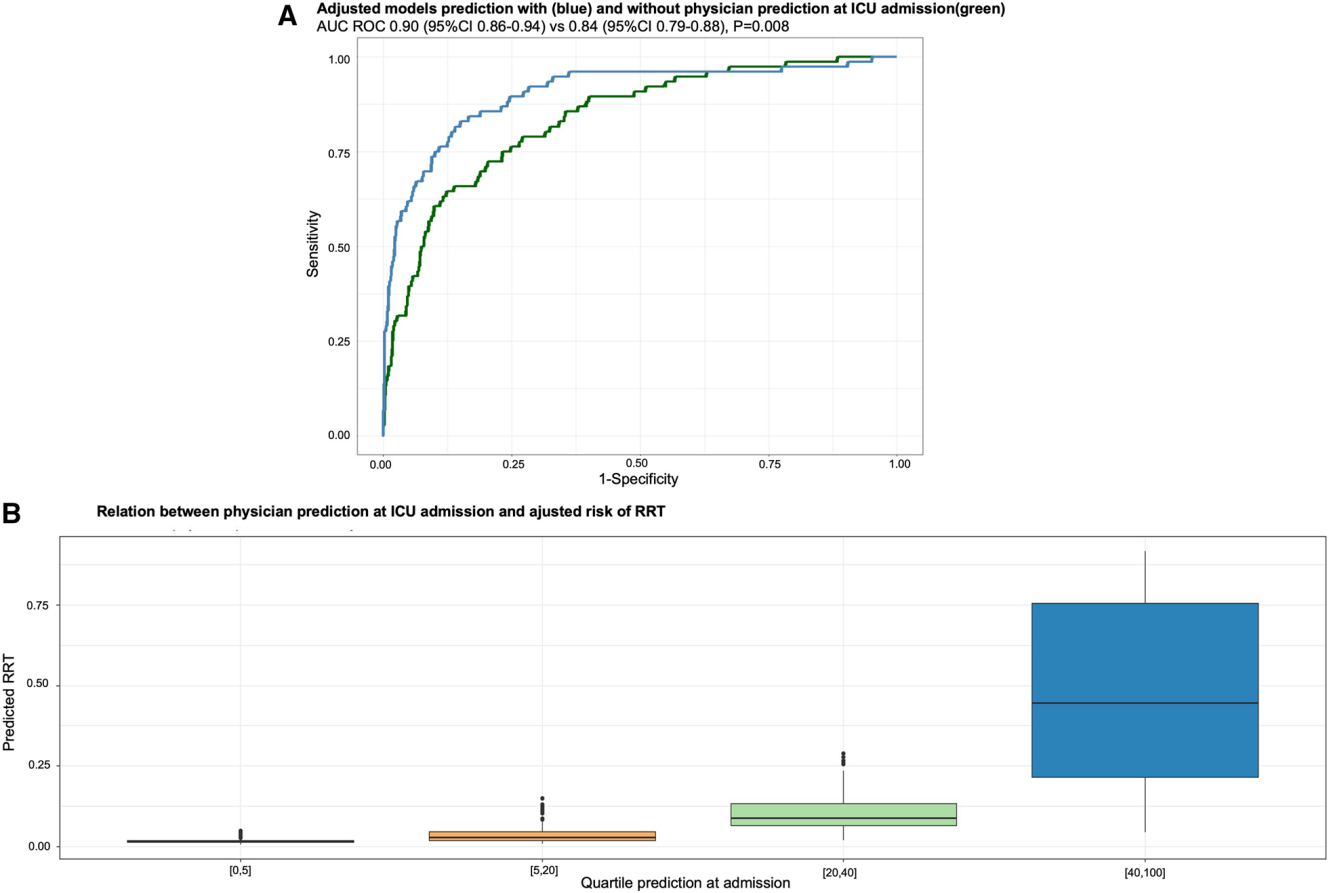
Adjusted models prediction of physician prediction at ICU admission (**A**) and relation between physician prediction and adjusted risk of RRT (**B**). *ICU*  intensive care unit, *RRT*  renal replacement therapy

In multivariate mixed model taking into account clustering by physician, a model including SOFA score, serum creatinine and diuresis at admission was selected and was able to predict the need for RRT during ICU stay with an AUC at 0.84 (95% CI 0.79–0.88) (Fig. 2A, Additional file 1: Table S4). After adjustment for these variables, physician prediction was maintained in the final model and strongly associated with the need for RRT (OR 1.06 per estimated % chance of receiving RRT; 95% CI 1.04–1.07, *p* < 0.0001) (Table 2). The relation between physician prediction at ICU admission and adjusted risk of RRT is reported in Fig. 2B.

**Table 2:** Physician’s prediction of need of RRT at ICU admission

Variables	OR [95% CI]	*P* value
SOFA score at admission	0.97 [0.89–1.06]	0.53
Serum creatinine—per 100 µmol/l	1.04 [0.87–1.25]	0.67
Urinary output at admission—ml/kg/h	0.85 [0.63–1.15]	0.30
Physician’s prediction at admission	1.06 [1.04–1.07]	< 0.001

*SOFA* Sequential Organ Failure Assessment, *ICU* intensive care units, *RRT *renal replacement therapy, *OR* odds ratio

A model including physician prediction, the experience of the physician, SOFA score, serum creatinine and diuresis to determine the need for RRT at ICU admission performed better than the model without physician prediction, with an AUC of 0.90 (95% CI 0.86–0.94, *p* < 0.008) (Fig. 2A). The implementation of the clinician prediction in our model resulted in an average performance improvement of 19.6% of the sensitivity and 3% of the specificity.

### Risk stratification of physician perception at AKI diagnosis

**Fig. 3:**
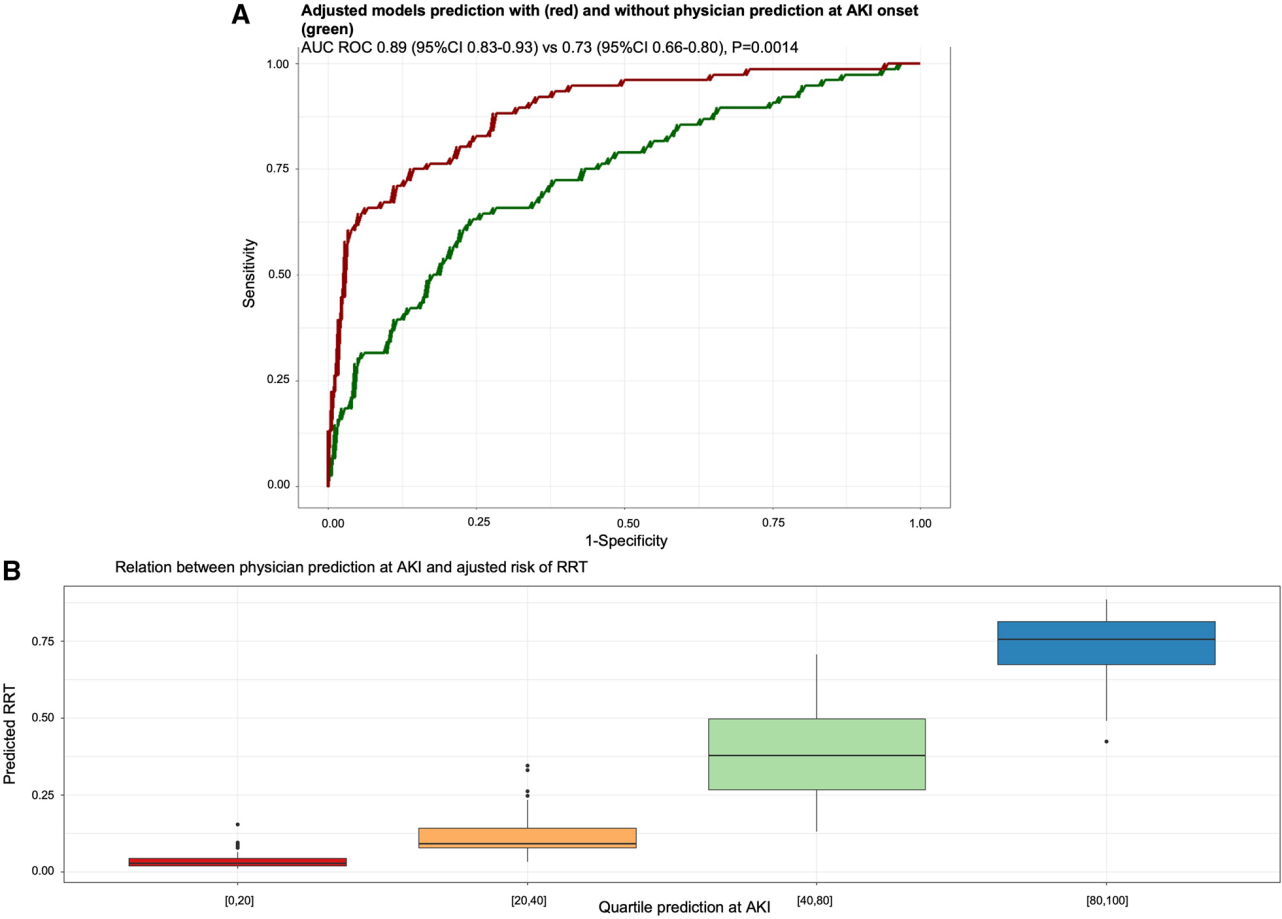
Adjusted models prediction of physician prediction at AKI diagnosis (**A**) and relation between physician prediction and adjusted risk of RRT (**B**)**.** *AKI* acute kidney injury, *RRT* renal replacement therapy

A model including the SOFA score, serum creatinine and diuresis was able to predict the need for RRT during ICU stay with an AUC at 0.73 (95% CI 0.66–0.80) (Fig. 3A). In multivariate analysis, after stepwise regression, physician prediction was maintained in the final model and strongly associated with the need for RRT (OR 1.06 per unit; 95% CI 1.04–1.07, *p* < 0.001), independently of creatinine levels, diuresis, SOFA score and the experience of the doctor who made the prediction (Table 3). The relation between physician prediction at AKI diagnosis and adjusted risk of RRT is shown in Fig. 3B.

**Table 3:** Physician’s prediction of need of RRT at AKI

Variables	OR [95% CI]	*P* value
SOFA score at AKI diagnosis	0.93 [0.85–1.02]	0.14
Serum creatinine—per 100 µmol/l	0.99 [0.85–1.17]	0.94
Urinary output at admission—ml/kg/h	0.94 [0.63–1.41]	0.76
Physician’s prediction at AKI diagnosis	1.06 [1.04–1.07]	< 0.001

*SOFA* Sequential Organ Failure Assessment, *AKI* acute kidney injury, *RRT* renal replacement therapy, *OR* odds ratio

A model including physician prediction, the experience of the physician, SOFA score, serum creatinine and diuresis to determine need for RRT at AKI diagnosis performed better than the model without physician prediction, with an area under the ROC curve of 0.89 (95% CI 0.83–0.93, *p* = 0.0014) (Fig. 3A). The implementation of the clinician prediction in our model resulted in an average performance improvement of 21.1% of the sensitivity and 8.9% of the specificity.

## Discussion

This is the first multicenter prospective study assessing the predicting performance of physicians to determine the need for RRT during ICU stay. Using a simple scale, we found a good correlation between clinician scores that were determined at ICU admission or at AKI diagnosis and the need for RRT. The implementation of clinician prediction improved the prediction of RRT requirement in ICU patients, at ICU admission and AKI diagnosis.

Subjective judgements of clinicians are difficult to evaluate and hard to compare. In order to quantify in a simple way physician’s prediction, we developed a simple tool using a 0 to 10 scale that showed good inter-rater reliability and improved the performance of our model to predict AKI outcomes. Edelson et al. have previously shown that clinical judgment regarding patient stability can be reliably quantified in a simple score, using a similar scale representing the likelihood of a patient experiencing a cardiac arrest or ICU transfer within the next 24 h [28]. Other studies have evaluated the accuracy of clinical judgment in predicting the need for mechanical ventilation or outcomes such as mortality in critically ill hospitalized patients [29].

These subjective judgments had good accuracy when compared to previously validated illness scoring systems, such as the Acute Physiology, Age, Chronic Health Evaluation (APACHE) system [30]. A meta-analysis of 12 observational studies which compared physician intuition to various physiologic scoring systems found that physicians discriminate between survivors and non-survivors more accurately than do scoring systems at ICU admission [31].

In a study focusing on the performance of clinicians to predict the duration of mechanical ventilation, Figueroa-Casas JB et al. found that the accuracy of intensivists' clinical predictions of duration of mechanical ventilation was limited with a raw agreement between predicted and actual durations, of 37% (CI 95% 29–45%) [32].

However, no study to date has specifically focused on the physician intuition to predict the need of RRT in ICU patients. Darmon et al*.* in a secondary analysis of a study focusing on the performance of Doppler-based renal resistive index to predict AKI outcomes found that the clinician’s estimation of the need for RRT was superior of Doppler-based renal resistive index with an AUC of 0.76 (95% CI 0.67–0.85) and an optimal cut-off of 75%, with a sensitivity of 63% (95% CI 49–77%) and a specificity of 77% (95% CI 72–81%) [14].

One recent study compared physician predication to determine development of AKI. In a single-center study including 252 patients at ICU admission, Flechet et al. compared the performance of the AKI risk estimated by physicians versus the one provided by a machine learning-based clinical prediction model [33]. They found that clinicians could predict AKI with good discrimination, but tend to overestimate the risk of AKI, pointing out a poor calibration in the low-risk patients. Although they found that the machine-learning based clinical prediction did better in terms of calibration and net benefit, only 30 (12%) patients developed AKI stage 2 and 3 in the first week of admission in this study.

In our study, 77 patients (28.5% of AKI patients) received RRT during ICU stay that allowed us to construct a robust predictive model including the best variables associated with RRT. We found that including physician prediction in our model was able to significantly improve the accuracy of the model. However, at an individual level, the prediction of the physician at ICU admission is insufficient to predict if one patient will experience RRT or not. Indeed, although the physician is able to stratify patients and discriminate patients at high or low risk of RRT requirement, we did not found a linear relationship between the estimation of the clinician and the need for RRT. If physicians may be good at detecting the need for RRT, that precision may decrease for prediction of lower stages of AKI. Indeed, Rank et al. in a study including patients after cardiothoracic surgery, showed that physicians underestimated the risk of AKI, especially stage 1 and 2 [34]. Moreover, our study was not designed to study the performance of the physicians to predict the risk of death of AKI patients, a prediction that may compete with the prediction of the risk of RRT.

Other studies have evaluated new approaches using machine learning to determine the need for RRT [35–38]. These machine learning scores combined with physician judgement may be useful tools to predict the need of RRT and design new randomized studies focusing on the timing of RRT in high-risk patients.

Previous studies have shown that clinician prediction performance for outcomes in hospitalized patients may vary according to clinical experience of the physician who complete the survey [28, 39]. In our multivariate model, the physician prediction was significantly associated with RRT requirement, independently of the clinical experience of the physician. All the participants of the study had at least 1 year of experience and 49 (47.5%) had > 5 years of experience that may explain our results.

AKIKI, IDEAL-ICU, and STARRT-AKI trials have shown that early dialysis for AKI did not confer any survival advantage [22, 40, 41]. More recently, AKIKI-2 compared the standard “delayed strategy” as employed in prior studies, and a more delayed strategy designed to postpone RRT initiation even longer [42]. Further delay in RRT did not show significant difference in RRT-free days or 60-day mortality between the two strategies. However, the multivariable analysis found that the 60-day mortality was higher with more delayed strategies. Identifying at ICU admission this specific subgroup of patients may be of importance in order to anticipate the need for RRT and start RRT before absolute indications in this population. In a heterogeneous group of pre-test probabilities, we cannot anticipate the treatment effect heterogeneity linked to this pre-test probability, i.e., how this pre-test probability may influence the decision to start RRT. Our results may then help to design new randomized studies focusing on new AKI treatment strategies in order to stratify patients before the randomization, taking into account the physician intuition at ICU admission.

This study has several limitations. First, although we ensured that the prediction for RRT requirement was performed by a physician who was not directly involved in the patient care, physicians who made the prediction and physicians in charge of the patient were part of the same team, rending difficult the complete independency between physician prediction and physician decision of RRT initiation. Second, in case of AKI, the physician assessment had been made the day of AKI diagnosis. Unfortunately, we did not have the precise hour in the day at which the AKI diagnosis and physician assessment were made. We cannot exclude that patient’s clinical course may have progressed during the day in the time between AKI diagnosis and physician assessment.

Third, as this study was observational, biomarkers were not available in our study, as most of the participating ICUs did not use biomarkers in routine. Although our results may suggest that our model is performant to predict AKI severity and RRT requirement without the use of these biomarkers, it is also possible that the performance of our model would have been improved by the use of such biomarkers.

Fourth, the questionnaire did not include the reasons behind physicians’ predictions.

Finally, in French ICUs, the decision to start RRT is made by the intensivist in charge of the patient. Our results may have been different in other settings, where the decision is left to an external nephrologist.

## Conclusion

The implementation of clinician prediction in a model evaluating the risk of RRT in critically ill patients improved the accuracy of the model, at ICU admission and AKI diagnosis. As clinicians are able, at different time points, to stratify patients at high risk of RRT, physician judgement should be taken into account when designing new randomized studies focusing on RRT initiation during AKI.

## Supplementary Information

**Additional file 1: Figure S1.** Physician prediction: Visual Likert Scale. **Figure S2.** PresagEER study timeline. **Table S1.** Delays between ICU admission, AKI diagnosis and RRT initiation. **Table S2.** RRT characteristics. **Table S3.** Characteristics and outcomes of AKI patients (n (%) or median (IQR)). **Table S4.** Multivariate analysis including variables associated with the risk of requiring RRT (without physician prediction).

## Data Availability

The data that support the findings of this study are available from the corresponding author, AS, upon reasonable request.

## Abbreviations

AKI: Acute kidney injury
RRT: Renal replacement therapy
IL-18: Interleukin-18
NGAL: Neutrophil gelatinase-associated lipocalin
KIM-1: Kidney injury molecule-1
TIMP-2: Tissue inhibitor of metalloproteinase-2
IGFBP7: Insulin-like growth factor-binding protein-7
ICU: Intensive care unit
SRLF: Société de réanimation de langue française
SOFA: Sequential Organ Failure Assessment
GFR: Glomerular filtration rate
CKD-EPI: Chronic kidney disease-epidemiology collaboration
ACEIs: Angiotensin-converting enzyme inhibitors
ARB: Angiotensin receptor blocker
NSAIDs: Non-steroidal anti-inflammatory drugs
CT scan: Computed tomography scan with intravenous iodine-based contrast media
ECMO: Extracorporeal membrane oxygenation
CVVHF: Continuous veno-venous hemofiltration
IHD: Intermittent haemodialysis
SLED: Sustained low-efficiency dialysis
KDIGO: Kidney disease improving global outcomes
MDRD: Modification of diet in renal disease
CI: Confidence interval
AUC: Area under the curve
