# Supplementary material for: Accuracy of clinicians’ ability to predict the need for renal replacement therapy: a prospective multicenter study
Source: Ann Intensive Care. 2022 Oct 15;12:95. doi: 10.1186/s13613-022-01066-w (PMC9569012; doi:10.1186/s13613-022-01066-w)
Supplement: Supplementary file 1 — Additional file 1: Figure S1. Physician prediction: Visual Likert Scale. Figure S2. PresagEER study timeline. Table S1. Delays between ICU admission, AKI diagnosis and RRT initiation. Table S2. RRT characteristics. Table S3. Characteristics and outcomes of AKI patients (n (%) or median (IQR)). Table S4. Multivariate analysis including variables associated with the risk of requiring RRT (without physician prediction). [file 13613_2022_1066_MOESM1_ESM.docx]

# Supplemental data


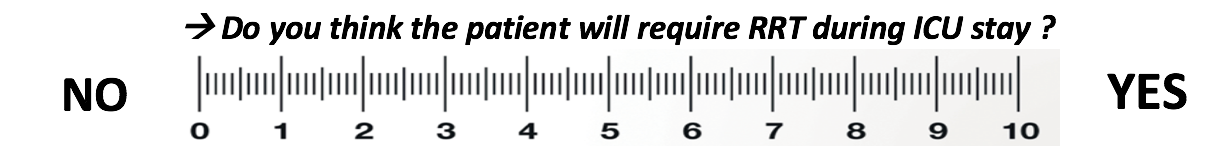


**Figure S1. Physician prediction : Visual Likert Scale** *(ICU = Intensive Care Unit ; RRT = Renal Replacement Therapy)*

0 : the physician is certain that the patient will not receive RRT

10 : the physician is certain that the patient will receive RRT

**
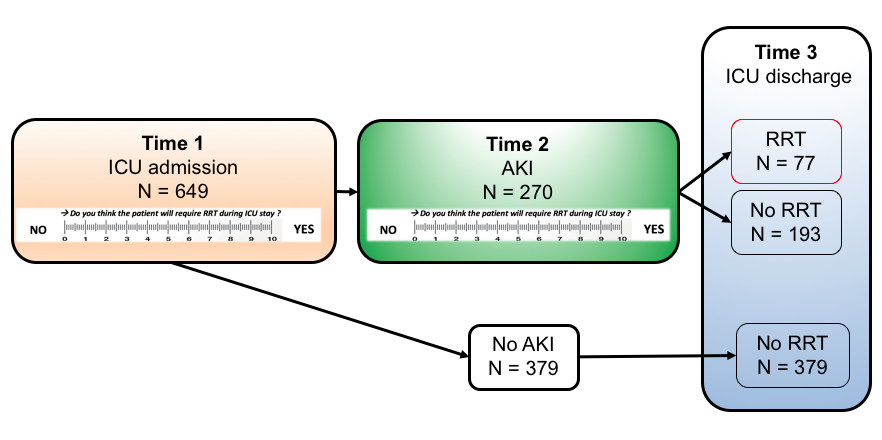
Table S1. Delays between ICU admission/AKI diagnosis/RRT initiation**

**Figure S2. PresagEER study timeline** *(ICU = Intensive Care Unit ; AKI = Acute Kidney Injury ; RRT = Renal Replacement Therapy)*

|  |  | AKI without RRT  (n=193) | AKI with RRT  (n=77) | AKI patients  (n=270) | P value |
| --- | --- | --- | --- | --- | --- |
| Delay between ICU admission and AKI diagnosis (n (%)) | **Days** |  |  |  |  |
|  | 0 | 107 (55.4) | 39 (50.6) | 146 (54.1) | 0,14 |
|  | 1 | 37 (19.2) | 12 (15.6) | 49 (18.1) |  |
|  | 2 | 19 (9.8) | 7 (9.1) | 26 (9.6) |  |
|  | 3 | 4 (2.1) | 4 (5.2) | 8 (3.0) |  |
|  | 4 | 5 (2.6) | 2 (2.6) | 7 (2.6) |  |
|  | 5 | 3 (1.6) | 2 (2.6) | 5 (1.9) |  |
|  | >5 | 18 (9.3) | 11 (14.2) | 29 (19.8) |  |
| Delay between AKI diagnosis and RRT initiation (n (%)) | **Days** |  |  |  |  |
|  | 0 |  | 29 (37.7) |  |  |
|  | 1 |  | 14 (18.2) |  |  |
|  | 2 |  | 3 (3.9) |  |  |
|  | 3 |  | 5 (6.5) |  |  |
|  | 4 |  | 1 (1.3) |  |  |
|  | 5 |  | 11 (14.3) |  |  |
|  | >5 |  | 14 (18.2) |  |  |

**Table S2. RRT characteristics**

| n (%) or Median (IQR) | RRT  (n=77) |
| --- | --- |
| Total duration of RRT | 4 [2, 10] |
| Initial RRT method  CRRT  IHD  SLED | 41 (53.2)  35 (45.5)  1 (1.3) |
| RRT dependency at ICU discharge | 49 (63.6) |
| Reasons for need of RRT  Hyperkalemia  Fluid overload  Uremic symptoms  Drug toxin  Electrolyte disorders  Metabolic acidosis  Tumor lysis  Hyperazotemia, oligo-anuria phase  Hyperthermia refractory | 20 (27.0)  30 (39.5)  9 (12.2)  7 (9.3)  13 (17.6)  31 (40.8)  4 (5.4)  41 (53.9)  0 (0) |
| Biological data at RRT initiation  Blood pH  Serum creatinine - µmol/l  Serum kalemia - mmol/l  Urinary output - ml/d  Urinary creatinine - mmol/l  Serum lactate - mmol/l  Serum bicarbonate - mmol/l  Serum phosphatemia - mmol/l | 7.29 [7.20, 7.36]  274 [202, 412]  4.6 [4.1, 5.6]  295 [3.15, 886]  4.6 [2.5, 6.5]  2.3 [1.2, 5.9]  19 [16, 23]  1.8 [1.2, 2.5] |
| Fluid balance - ml/d (RRT initiation) | 1250 [381, 3068] |
| *RRT = Renal Replacement Therapy ; CRRT = Continuous Renal Replacement Therapy ; IHD = Intermittent Hemodialysis ; SLED = Sustained Low-Efficiency Dialysis ; ICU = Intensive Care Unit* | |

**Table S3. Characteristics and outcomes of AKI patients (n (%) or median (IQR))**

| Characteristics | AKI without RRT  (n=193) | AKI with RRT  (n=77) | AKI patients  (n=270) | P value |
| --- | --- | --- | --- | --- |
| Age years | 66 [57, 74] | 64 [55, 71] | 66 [56, 73] | 0.14 |
| Gender | 44 (22.8) | 20 (26.0) | 64 (23.7) | 0.69 |
| BMI | 26 [23, 30] | 28 [24, 34] | 27 [23, 31] | 0.008 |
| CCI | 4 [3, 7] | 4 [3, 6] | 4 [3, 6] | 0.87 |
| SOFA score at AKI diagnosis | 8 [4, 11] | 10 [6, 13] | 8 [5, 11] | 0.001 |
| Comorbidity  CKD  Congestive heart failure  Myocardial infarction  Diabetes mellitus  Peripheral vascular disease  Chronic pulmonary disease  Connective tissue disease  Liver disease  Hematological disease  Metastatic solid tumor  AIDS | 39 (20.2)  33 (17.1)  36 (18.7)  58 (30.1)  25 (13.9)  40 (20.7)  2 (1.1)  27 (14.0)  11 (5.7)  9 (5.0)  5 (2.8) | 18 (23.4)  15 (19.7)  9 (11.7)  27 (35.1)  13 (17.6)  10 (13.0)  3 (4.1)  16 (20.8)  8 (10.4)  3 (4.1)  3 (4.1) | 57 (21.1)  48 (17.8)  45 (16.7)  85 (31.5)  38 (15.0)  50 (18.5)  5 (2.0)  43 (15.9)  19 (7.0)  12 (4.7)  8 (3.2) | 0.68  0.74  0.23  0.51  0.58  0.19  0.30  0.23  0.27  1.00  0.90 |
| ICU : reasons for admission  Medical  Elective surgery  Emergency surgery | 184 (95.3)  3 (1.6)  6 (3.1) | 71 (92.2)  1 (1.3)  5 (6.5) | 255 (94.4)  4 (1.5)  11 (4.1) | 0.44 |
| Physician’s ICU experience  <2 years  [2-5] years  [5-10] years  >10 years | 49 (25.4)  51 (26.4)  48 (24.9)  45 (23.3) | 29 (37.7)  13 (16.9)  15 (19.5)  20 (26.0) | 78 (28.9)  64 (23.7)  63 (23.3)  65 (24.1) | 0.12 |
| Physician’s ICU seniority  Fellows  Attendings | 100 (51.8)  93 (48.2) | 42 (54.5)  35 (45.5) | 142 (52.6)  128 (47.4) | 0.79 |
| KDIGO score staging  I  II  III | 93 (48.2)  54 (28.0)  46 (23.8) | 21 (27.3)  21 (27.3)  35 (45.5) | 114 (42.2)  75 (27.8)  81 (30.0) | 0.001 |
| Time from admission to AKI diagnosis – days | 0 [0, 2] | 0 [0, 3] | 0 [0, 2] | 0.36 |
| Data’s at AKI diagnosis  Serum creatinine  Urinary Output - mlkgh  Fluid balance - ml/d  Fluid balance - ml/kg/h | 151 [119, 212]  0.5 [0.3, 0.8]  507 [-189, 1876]  0.1 [0, 0.7] | 209 [120, 261]  0.3 [0.1, 0.6]  884 [88, 2543]  0.3 [0, 1] | 160 [119, 233]  0.4 [0.2, 0.8]  596 [-35, 2000]  0.1 [0, 0.8] | 0.03  <0.001  0.07  0.03 |
| Renal ultrasonography | 67 (34.9) | 25 (32.5) | 92 (34.2) | 0.81 |
| AKI causes  Nephrotoxic use  Obstructive cause  Prerenal causes  Intrinsic renal causes | 52 (26.9)  7 (3.6)  154 (79.8)  50 (25.9) | 38 (49.4)  0 (0.0)  62 (80.5)  42 (54.5) | 90 (33.3)  7 (2.6)  216 (80.0)  92 (34.1) | 0.001  0.20  1.00  <0.001 |
| Events week before AKI  Coronary angiography  Mechanical ventilation  Vasopressor use  CT scan  ECMO  Sepsis | 5 (2.6)  80 (41.7)  84 (43.5)  85 (44.3)  2 (1.0)  90 (46.9) | 4 (5.3)  47 (61.8)  46 (60.5)  30 (39.5)  4 (5.3)  45 (59.2) | 9 (3.4)  127 (47.4)  130 (48.7)  115 (42.9)  6 (2.2)  135 (50.4) | 0.48  0.004  0.02  0.56  0.10  0.09 |
| Physician's prediction to need of RRT | 3 [1, 6] | 9 [6, 10] | 4 [2, 8] | <0.001 |
| Outcomes  ICU Mortality  COVID (%)  ICU duration (median [IQR]) | 52 (26.9)  64 (33.2)  5 [3, 11] | 48 (62.3)  30 (39.0)  13 [5, 21] | 100 (37.0)  94 (34.8)  7 [3, 15] | <0.001  0.446  <0.001 |
| *AKI = Acute Kidney Injury ; BMI = Body Mass Index ; CCI = Charlson Comorbidity Index ; SOFA = Sequential Organ Failure Assessment ; CKD = Chronic Kidney Disease ; AIDS = Acquired Immuno Deficiency Syndrome ; ICU = Intensive Care Unit ; RRT = Renal Replacement Therapy , KDIGO = Kidney Disease: Improving Global Outcomes ; CT scan = Computed Tomography scanner ; ECMO = Extracorporeal Membrane Oxygenation* | | | | |

**Table S4. Multivariate analysis including variables associated with the risk of requiring RRT (without physician prediction)**

| Variables | OR [95% CI] | P value |
| --- | --- | --- |
| SOFA score at ICU admission | 1.16 [1.09-1.24] | < 0.001 |
| Serum Creatinine - per 100µmol/l | 1.48 [1.17-1.87] | <0.01 |
| Urinary Output at ICU admission - mg/kg/h | 0.74 [0.54-1.03] | 0.07 |
| *SOFA = Sequential Organ Failure Assessment ; AKI = Acute Kidney Injury ; RRT = Renal Replacement Therapy ; OR = Odds Ratio* | | |
